# sFRP3 inhibition improves age‐related cellular changes in BubR1 progeroid mice

**DOI:** 10.1111/acel.12899

**Published:** 2019-01-04

**Authors:** Chang Hoon Cho, Ki Hyun Yoo, Alfredo Oliveros, Summer Paulson, Syed Mohammed Qasim Hussaini, Jan M. van Deursen, Mi‐Hyeon Jang

**Affiliations:** ^1^ Department of Neurologic Surgery Mayo Clinic Rochester Minnesota; ^2^ SURF program, Neuroscience Track, Mayo Clinic Graduate School of Biomedical Sciences Rochester Minnesota; ^3^ Currently at Duke University Medical Center Durham North Carolina; ^4^ Department of Biochemistry and Molecular Biology Mayo Clinic Rochester Minnesota; ^5^ Department of Pediatric and Adolescent Medicine Mayo Clinic Rochester Minnesota

**Keywords:** BubR1, myelination, neurogenesis, progeria, sFRP3

## Abstract

Wnt signaling is a well‐known molecular pathway in age‐related pathogenesis and therapy of disease. While prior studies have mainly focused on Wnt ligands or Wnt activators, the in vivo functions of naturally secreted Wnt inhibitors are not clear, especially in brain aging. Using *BubR1* ^H/H^ mice as a novel mouse model of accelerated aging, we report that genetic inhibition of sFRP3 restores the reduced body and brain size observed in *BubR1* ^H/H^ mice. Furthermore, sFRP3 inhibition ameliorates hypomyelination in the corpus callosum and rescues neural progenitor proliferation in the hippocampal dentate gyrus of *BubR1* ^H/H^ mice. Taken together, our study identifies sFRP3 as a new molecular factor that cooperates with BubR1 function to regulate brain development, myelination, and hippocampal neurogenesis.

## 1 INTRODUCTION

Decline in the mitotic checkpoint kinase BubR1 level occurs with natural aging and induces progeroid features in mice and humans with mosaic variegated aneuploidy syndrome (Baker et al., 2004). Our previous studies show that BubR1 expression levels in WT mice significantly decline with natural aging in the brain. Mutant mice producing low levels of BubR1 (*BubR1* ^H/H^ mice) exhibit smaller brain sizes and were rescued by constitutive overexpression of BubR1, suggesting that brain development is mediated through BubR1 (Supporting Information Figure S1). In addition, *BubR1* ^H/H^ mice exhibit reduced hippocampal neurogenesis and impaired myelination (Choi et al., 2016; Yang et al., 2017). Together, these findings suggest that decreased BubR1 expression with aging contributes to brain dysfunction. Therefore, determining a molecular target that can counteract this process is of great interest.

We previously showed that a genetic deletion of secreted frizzled related protein 3 (sFRP3), an endogenous Wnt antagonist, stimulated adult hippocampal neurogenesis (Jang, Bonaguidi, et al., 2013) and promotes antidepressant action in mice and humans (Jang, Kitabatake, et al., 2013). While enhancing Wnt signaling can ameliorate age‐related deficits in cellular and cognitive function (Seib et al., 2013), we sought to determine whether inhibition of sFRP3 also has a neuroprotective role in BubR1‐regulated brain aging.

**Figure 1:**
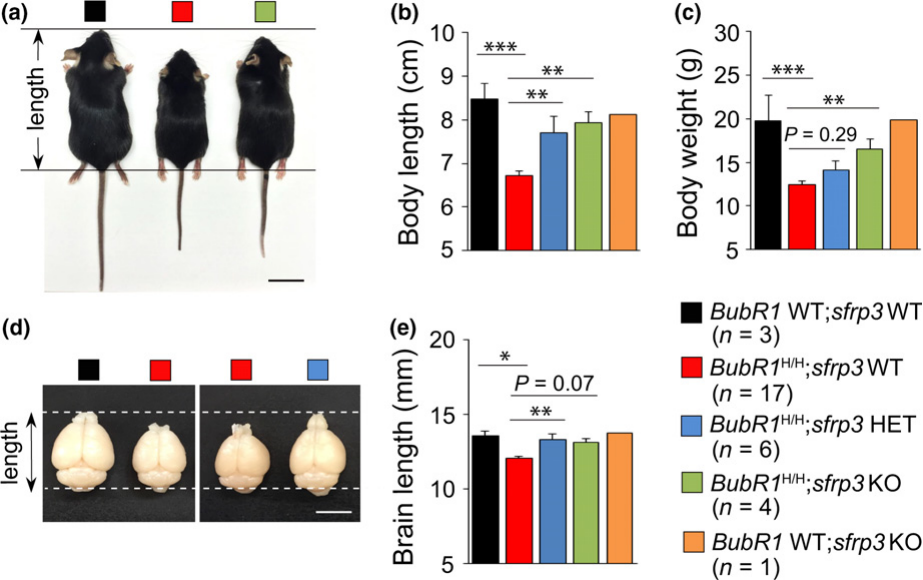
Genetic inhibition of sFRP3 restores body and brain size of *BubR1* ^H/H^ mice. (a–c) Representative images and quantification of body size and body weight of each group. Scale bar: 1 cm. KO: knockout; HET: heterozygous. (d, e) Representative images and quantification of brain size of each group. Scale bar: 0.5 cm. All values represent mean ± *SEM*. (^*^ *p* < 0.05, ^**^ *p* < 0.01, ^***^ *p* < 0.001, one‐way ANOVA)

To address this in vivo, *sfrp3* knockout (KO) mice were crossed to *BubR1* ^H/H^ mice. In comparison with *BubR1* ^H/H^;*sfrp3* WT mice, which displayed significant decreases in body and brain size, we found that both *BubR1* ^H/H^;*sfrp3* KO mice and *BubR1* ^H/H^;*sfrp3* HET mice exhibited restored body (Figure 1a–c) and brain size (Figure 1d,e). These results indicate an importance of sFRP3 in this process.

**Figure 2:**
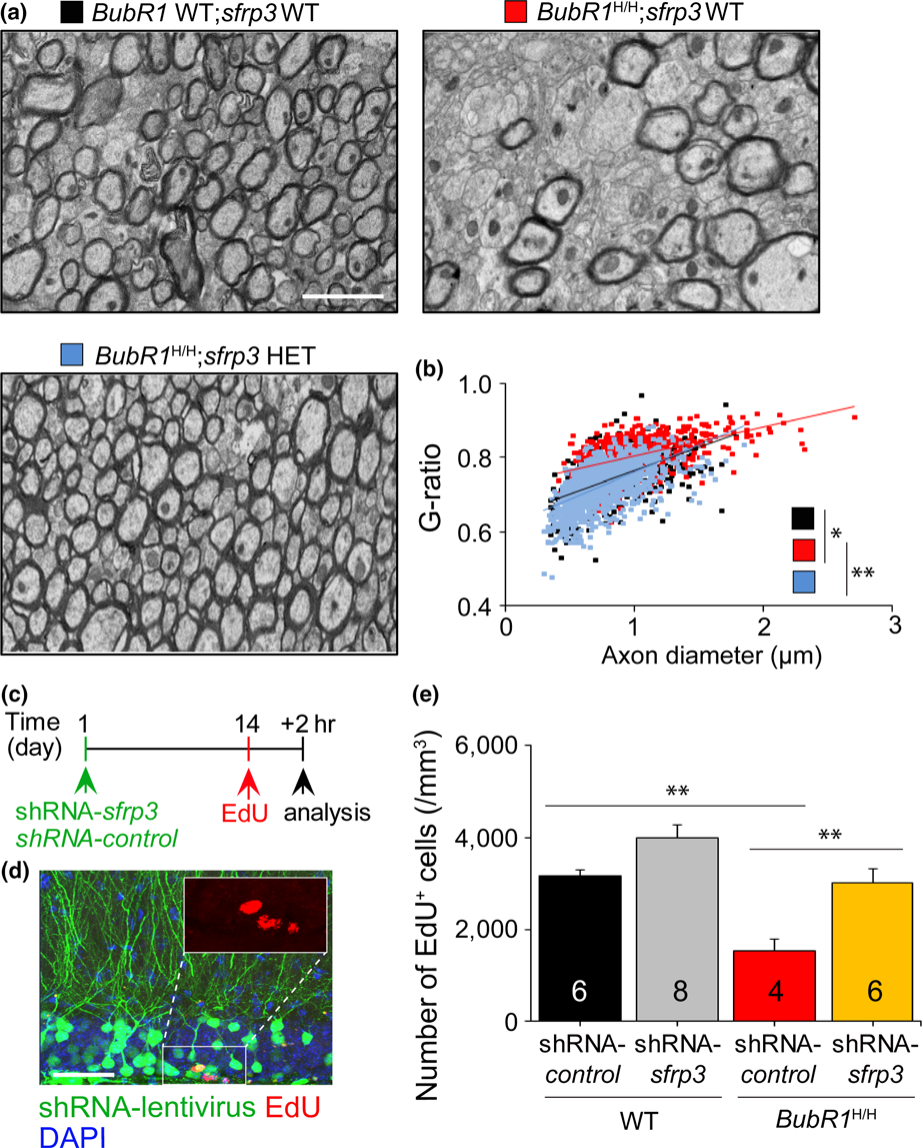
Genetic inhibition of sFRP3 ameliorates cellular abnormalities observed in *BubR1* ^H/H^ mice. (a) Electron microscopy analysis of the corpus callosum of each genotype. Scale bar: 2 µm. (b) Scatter diagram and quantification of G‐ratio. (c) Experimental design. (d) Representative confocal images demonstrating infected lentiviral tdTomato^+^ (green), EdU^+^ (red; inset), and DAPI^+^ (blue) cells. Scale bar: 20 μm. (e) Quantification of EdU^+^ cells in each group. All values represent mean ± *SEM*. (^*^ *p* < 0.05, ^**^ *p* < 0.01, ^***^ *p* < 0.001, two‐way ANOVA). Number associated with bar graphs indicates number of mice tested

How sFRP3 inhibition restores reduced brain size in *BubR1* ^H/H^ mice is unknown. In our previous report, we showed that *BubR1* ^H/H^ mice exhibited smaller brain sizes mainly attributed to hypomyelination, resulting in abnormal corpus callosum formation (Choi et al., 2016). Consequently, we tested whether sFRP3 inhibition could ameliorate impaired myelination in *BubR1* ^H/H^ mice. Our examination of myelin density (Supporting Information Figure S2) and ultrastructural analysis (Figure 2a,b) revealed that *BubR1* ^H/H^;*sfrp3* WT mice showed profound hypomyelination in the adult corpus callosum. However, *BubR1* ^H/H^;*sfrp3* HET or *BubR1* ^H/H^;*sfrp3* KO mice show significantly improved myelination compared to *BubR1* ^H/H^;*sfrp3* WT mice, indicating that genetic inhibition of sFRP3 prevents hypomyelination, potentially explaining how sFRP3 inhibition normalizes reduced brain size in *BubR1* ^H/H^ mice.

Aging is known to result in significant reductions in hippocampal neurogenesis and cognitive dysfunction. We previously reported that *BubR1* ^H/H^ mice exhibited impaired hippocampal neurogenesis (Yang et al., 2017). Therefore, we examined whether inhibition of sFRP3 could mitigate reduced neurogenesis in *BubR1* ^H/H^ mice. To test sFRP3’s specific role in the dentate gyrus, we used lentiviruses to acutely knockdown the expression of endogenous sFRP3 with short‐hairpin RNA (shRNA) in a non‐cell autonomous manner (Figure 2c,d). Stereological analysis showed a significant decrease in EdU^+^ cell density in the subgranular zone (SGZ) of shRNA‐control injected *BubR1* ^H/H^ mice compared to shRNA‐control injected WT mice. However, shRNA‐*sfrp3* injected *BubR1* ^H/H^ mice showed significantly increased EdU^+^ cell density compared to shRNA‐control injected *BubR1* ^H/H^ mice (Figure 2e), suggesting that sFRP3 knockdown ameliorates impaired neural progenitor proliferation in *BubR1* ^H/H^ mice.

In this study, we were initially surprised by the ability of sFRP3 inhibition to reverse the microcephaly phenotype. Wnt signaling serves a prominent role in the regulation of early brain development, neurogenesis, cell migration, dendrite morphogenesis, and synapse formation, as well as cognitive function. Accordingly, Wnt signaling has been a therapeutic target for a diverse range of neurodevelopmental, neurological and neurodegenerative disorders (Hussaini et al., 2014). While prior studies have mainly focused on Wnt ligands or Wnt activators in disease pathogenesis and therapy, recent discoveries focusing on the naturally secreted Wnt inhibitors have just started to shed light on it. For example, the Wnt antagonists such as Dickkopf‐1 (Dkk1) or Dkk3 are known to increase with age. Conditional deletion of Dkk1 enhances neurogenic function to counteract cognitive deficits (Seib et al., 2013; Zhu et al., 2014). Interestingly, different from these Wnt antagonists, hippocampal sFRP3 mRNA levels were unchanged across the lifespan (Supporting Information Figure S3a), suggesting that sFRP3‐mediated Wnt inhibition is not a contributing factor in age‐related neuropathology. Rather, sFRP3 reduction stimulates adult hippocampal neurogenesis (Jang, Bonaguidi, et al., 2013) and neuroprotective promotion of myelination, as we demonstrate that sFRP3 deletion significantly upregulates multiple genes critical for myelin production including MBP (Supporting Information Figure S3b). Thus, we propose that mechanistically, inhibition of sFRP3 rescues *BubR1* ^H/H^‐mediated deficits in neural progenitor proliferation, myelination, and brain growth.

Abnormalities in myelination and neurogenesis are hallmarks of age‐related neurodegeneration. Therefore, the ability to sustain myelin integrity and neurogenesis holds significant implications for aging, age‐related disorders, and future therapeutic strategies. In this regard, identification of sFRP3 function provides a novel perspective on the development of effective therapies preventing cognitive decline associated with neurodevelopmental and age‐related disorders.

## CONFLICT OF INTEREST

The authors declare no competing interests.

## AUTHOR CONTRIBUTIONS

C.H.C., K.H.Y., and M.H.J. designed research; C.H.C., K.H.Y., S.P., S.M.Q.H., and A.O. performed research; J.M.v.D. provided BubR1^H/H^ and BubR1^T23^ overexpression mice; C.H.C., K.H.Y., S.M.Q.H., A.O., and M.H.J. wrote the paper.
