# Supplementary material for: sFRP3 inhibition improves age‐related cellular changes in BubR1 progeroid mice
Source: Aging Cell. 2019 Jan 4;18(2):e12899. doi: 10.1111/acel.12899 (PMC6413750; doi:10.1111/acel.12899)
Supplement: Supplementary file 1 [file ACEL-18-e12899-s001.docx]

**Supporting Information**

**SFRP3 inhibition improves age-related cellular changes in BubR1 progeroid mice**

Chang Hoon Cho, Ki Hyun Yoo, Alfredo Oliveros, Summer Paulson, Syed Mohammed Qasim Hussaini, Jan M. van Deursen, Mi-Hyeon Jang

**Supporting Information includes**

Detailed materials and methods

**Fig. S1.** BubR1 regulates brain growth

**Fig. S2.** Inhibition of sFRP3 reverses hypomyelination

**Fig. S3.** Genetic deletion of sFRP3 promotes myelin-related genes

**Materials and Methods**

**Mice and breeding strategy**

*BubR1*^H/H^ mice were kindly provided by Dr. Jan M. van Deursen at Mayo Clinic, and sFRP3 KO mice were provided by Dr. Jeremy Nathans at Johns Hopkins University. BubR1 hypomorphic (*BubR1*^H/H^) and sFRP3 KO mice were generated as previously described ([Jang *et al.* 2013](#_ENREF_3); [Yang *et al.* 2017](#_ENREF_4)). *BubR1*^H/H^ mice were backcrossed to the C57BL/6 background for over 10 generations. Briefly, adult *BubR1* HET (*BubR1*^H/+^) were crossed to sFRP3 HET mice to generate *BubR1* HET;sFRP3 HET mice. *BubR1* HET;sFRP3 HET mice were then interbred to *BubR1* HET;sFRP3 HET mice to generate *BubR1* WT;sFRP3 WT mice, *BubR1*^H/H^;sFRP3 WT mice, *BubR1*^H/H^;sFRP3 HET mice, and *BubR1*^H/H^;sFRP3 KO mice for brain and body size as well as myelination analysis. To ensure that sFRP3 deletion alone does not have any detrimental impact on gross body and brain morphologies due to developmental consequences arising from embryonic sFRP3 deletion, we compared gross body and brain morphology in *BubR1* WT;sFRP3 WT group, and *BubR1* WT;sFRP3 KO group. Consistent with observations from our previous finding ([Jang *et al.* 2013](#_ENREF_3)), there is no effect on gross body and brain morphology in *BubR1* WT;sFRP3 KO group. However, because breeding difficulties resulted in low numbers of *BubR1* WT;sFRP3 KO mice available for experiments, we only include one animal. We should note that we do not directly compare this sFRP3 KO group with other groups. For all experiments, female mice were utilized, unless otherwise specified. All groups of mice at 8-10 weeks of age were used at the time of initiating experiments. *BubR1* WT;sFRP3 WT mice served as controls. Mice were housed in standard cages under a 12 h light/dark cycle with lights on at 7:00 AM. Food and water were provided *ad libitum*. All experiments involving animals were carried out in accordance with National Institutes of Health guidelines and were approved by the Mayo Clinic Institutional Animal Care and Use Committee (IACUC).

**Electron microscopy and myelin ultrastructural analysis**

The thickness of myelin sheaths was defined by ultrastructural analysis of the corpus callosum of the mouse brain at postnatal day 56. Electron microscopy was performed at the Electron Microscopy Core at Mayo Clinic. Briefly, mice were perfused with Trump’s fixative (4% formaldehyde with 1% glutaraldehyde, pH 7.4), followed by overnight post-fixation of the brains in the same solution. The corpus callosum brain tissue was excised, cut to 1 mm thickness in at least one dimension and placed in McDowell's and Trump's fixative for 1 hour at 4º C. Following 2 rinses in 0.1M sodium phosphate buffer (pH 7.2), samples were placed in 1% osmium tetroxide in the same buffer for 1 hour at room temperature. Samples were rinsed 2 times in distilled water and dehydrated in an ethanolic series culminating in two changes of 100% acetone. Tissue samples were then placed in a mixture of Spurr (2) resin and acetone (1:1) for 30 min, followed by 2 hours in 100% resin with 2 changes. Samples were then placed in fresh 100% resin in molds and polymerized at 65º C for 12 hours. Semi-thin (0.25-0.5 um) sections were cut, placed on glass slides and stained with 1% toluidine blue-O in 1% sodium borate. Ultrathin (70-90 nm) sections were cut with a diamond knife, stained with lead citrate and examined with a JEM-1400 Transmission Electron Microscope (JEOL USA, Peabody, MA). Images were randomly captured at 10000X without knowledge of genotype. G-ratio was calculated from all myelinated axons in each image. Measurements of axon diameter (d) and myelin fiber diameter (D) were made by including axons of all diameter with NIH ImageJ software using G-ratio plug-in and presented as mean G-ratio (d/D; SFig. 2C) as we used previously ([Choi *et al.* 2016](#_ENREF_2)).

**Luxol fast blue staining**

Luxol fast blue staining for myelin was carried out to evaluate the extent of myelination ([Choi *et al.* 2016](#_ENREF_2)). Brain sections underwent defattening, followed by immersion in 0.1% Luxol fast blue solution at 37ºC for 2 days, and 95% ethanol for 5 min. These were then incubated for 1 min in 0.05% lithium carbonate solution, and washed using 70% ethanol and distilled water respectively. Slices were sealed for microscopic observation.

**Construction and lentiviral production**

To effectively knockdown of sFRP3 selectively within the adult mouse dentate gyrus, we utilized previously generated shRNA-*sfrp3* or shRNA-control lentiviral constructs, which has been shown to have 75% knockdown efficiency ([Jang *et al.* 2013](#_ENREF_3)). Briefly, a lentiviral vector pFUGW co-expressing shRNA under the U6 promoter and tdTomato under the EF1α promoter were used. The short hairpin sequences used are as follows: (shRNA-*sfrp3*) 5’-GCTAGCGATTCCACTCAGAAT-3’; (shRNA-control) 5’-AGTTCCAGTACGGCTCCAA-3’. Lentiviruses were produced by co-transfection of lentiviral vectors and VSVG into HEK293T cells followed by ultra-centrifugation of viral supernatant. As shown in Fig. 2C, highly concentrated lentiviruses co-expressing shRNA-*sfrp3* or shRNA-control and tdTomato were stereotaxically injected into the dentate gyrus of 8-10 week-old *BubR1*^H/H^ mice and their WT littermates under the anesthesia at 4 sites (0.5 µl per site at 0.25 µl/min) with the following coordinates (in mm): posterior = -2 from Bregma, lateral = ± 1.6, ventral = 2.2; posterior = 3 from Bregma, lateral = ± 2.6, ventral = 3.2. At 14 days post-viral injection, mice were injected with EdU (41.1 mg/kg body weight, *i.p.*) to label dividing cells, and transcardially perfused 2 hours later with cold PBS followed by 4% paraformaldehyde solution. To confirm non-cell autonomous effects of shRNA-mediated lentiviral strategy, we assessed number of EdU-labeled cells that were also positive for lentiviral labeling, and found very minimal overlap (5.1 ± 2.05%; n = 4 animals), which further strengthens our model stating that sFRP3 regulates neurogenesis in a non-cell autonomous mechanism.

**EdU labeling, immunohistochemistry, confocal imaging, and analysis**

After post-fixation and cryoprotection, coronal brain sections (40 µm in thickness) were made in serial order for a total of ~50 sections along the anterior-posterior axis using a microtome (Leica SM 2010R). Approximately 4-5 brain sections in each group were obtained in serial order of dentate gyrus from the anterior to posterior axis, and processed for immunostaining as previously described ([Jang *et al.* 2013](#_ENREF_3)). EdU staining, presented in Fig. 2D, was performed using a Click-iT™ EdU Cell Proliferation Assay Kit with Alexa-Fluor 488 (Invitrogen) following the manufacturer's instructions ([Chehrehasa *et al.* 2009](#_ENREF_1); [Yang *et al.* 2017](#_ENREF_4)). For quantification, all z-stack images in entire dentate gyrus were acquired on a Zeiss LSM 780 single-photon confocal system using a multi-track configuration. Stereological quantification of EdU^+^ cells in the subgranular zone (SGZ) were performed as described previously ([Yang *et al.* 2017](#_ENREF_4)). Briefly, EdU^+^ cells along the SGZ were counted to obtain an absolute cell number. In each of the counted dentate gyri, the dentate gyrus area was measured from the middle z-plane, by manually tracing along its edge and enclosing it using the area measurement feature provided by the Zen software (Zeiss). The volume of the dentate gyrus section was calculated by multiplying the area by its thickness. The cell count was divided by the resultant section volume to obtain the total cell density in the dentate gyrus per mm^3^.

**Quantitative RT-PCR (qRT-PCR)**

For qRT-PCR, presented in SFig. 3, total RNA was isolated from mouse hippocampal tissues using the TRIzol® Reagent (Life techenologies). cDNA was generated using SuperScript III Reverse Transcriptase (Invitrogen) following the manufacturer’s protocol, and qRT-PCR was performed using a Quantstudio 3 Real-Time PCR System (Applied Biosystems) with Fast SYBR Green Master Mix (Applied Biosystems). Briefly, RNA was initially denatured for 5 min followed by 40 cycles of denaturing at 95°C for 15 sec, and annealing/elongation at 60°C for 1 min. All primer sequences used in this study are listed in Supplementary Table 1.

**Statistics**

All statistical analysis was performed using GraphPad Prism 7.00. One or two-way ANOVA was performed with Dunnetts or Tukey *post-hoc* test for multiple comparisons. One outlier on Fig. 2E was identified *via* GraphPad Prism QuickCalcs Grubbs outlier test, and this value was removed. Statistical significance was defined as *P* < 0.05.

**Supplementary Figures**

**
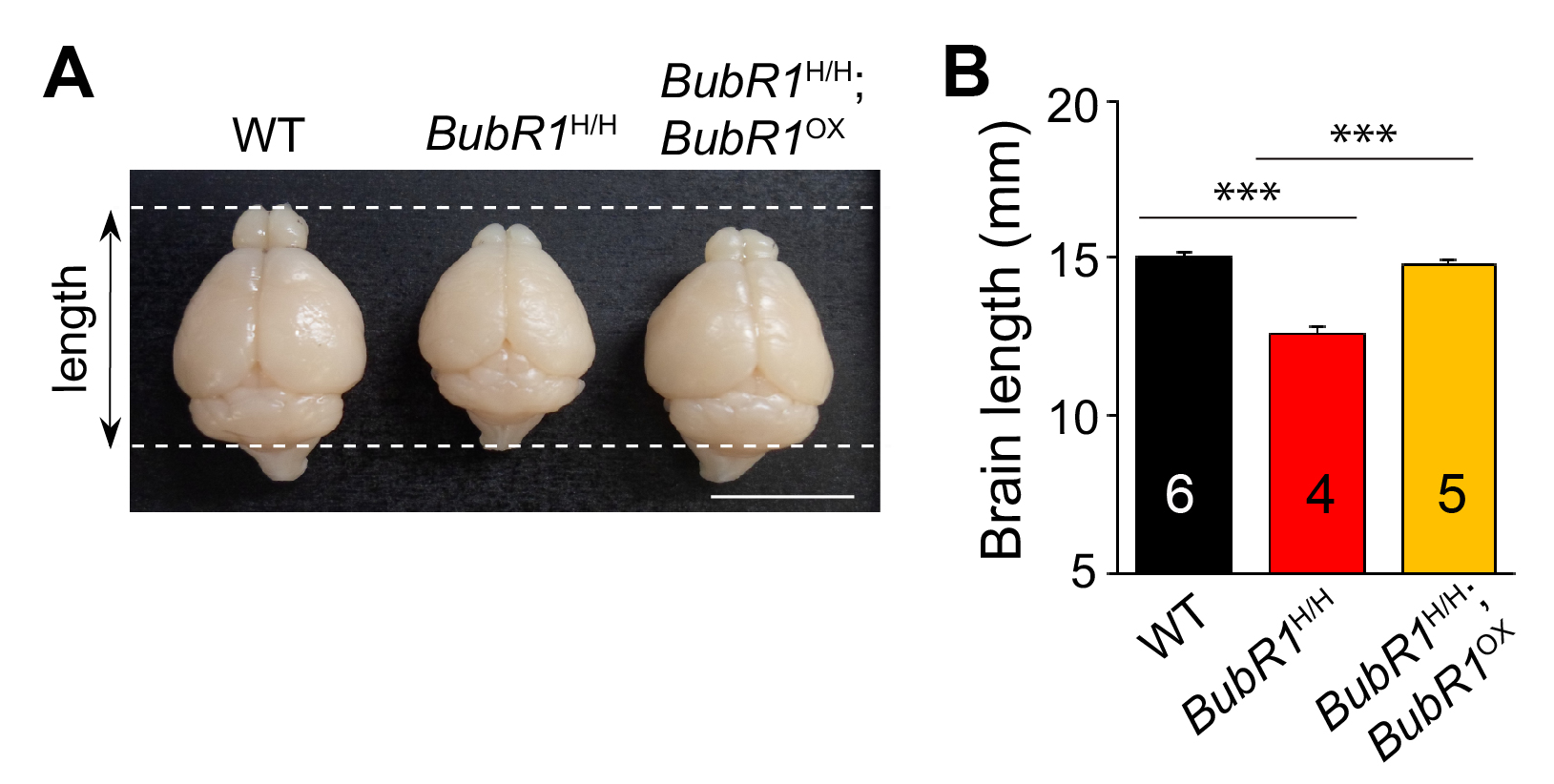
**

**SFig. 1. BubR1 regulates brain development**

**(A)** Representative images of brain size of *BubR1*^WT^ mice, *BubR1*^H/H^ mice, and *BubR1*^H/H^;BubR1 overexpression (OX; *BubR1*^T23^ line) mice. Scale bar: 1 cm. **(B)** Quantification of brain length in each group. Note that the small brain size observed in *BubR1*^H/H^ mice was rescued by BubR1 overexpression, indicating that brain formation is mediated through BubR1. All values represent mean ± SEM (***: *P* < 0.001, One-way ANOVA). The number associated with each bar graph represents the number of animals examined.

**
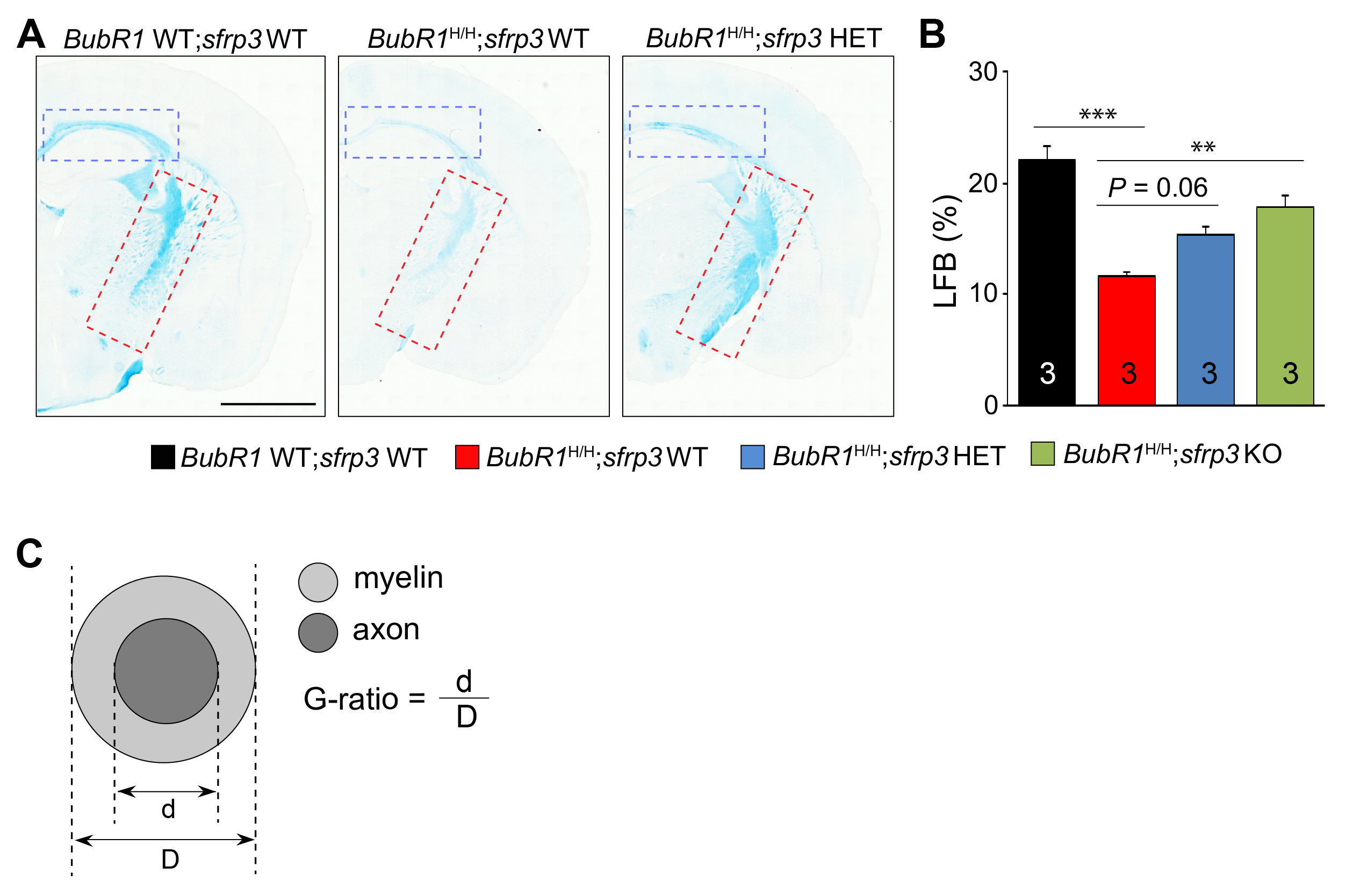
**

**SFig. 2. Inhibition of sFRP3 reverses hypomyelination**

(**A-B**) Luxol fast blue (LFB) staining analysis. (**A**) Representative images of LFB staining in each group. Scale bar: 2 mm. (**B**) Quantification of LFB area in coronal brain sections of 8-week-old *BubR1*^H/H^ mice exhibit a profound reduction in myelin density in the corpus callosum (blue dashed box), and internal capsule (red dashed box). Notably, genetic inhibition of sFRP3 (*BubR1*^H/H^;*sfrp3* HET or *BubR1*^H/H^;*sfrp3* KO mice) ameliorates reduced myelin density. All values represent mean ± SEM (**: *P* < 0.01, ***: *P* < 0.001; One-way ANOVA). Number associated with bar graphs indicates number of animals examined. (**C**) The G-ratio, an indicator of demyelination defined as the ratio of the diameter of the axon to the diameter of the axon plus the surrounding myelin (the formula is shown in the insert), shows that the increased G-ratio in the *BubR1*^H/H^ mice was abolished by genetic inhibition of sFRP3.


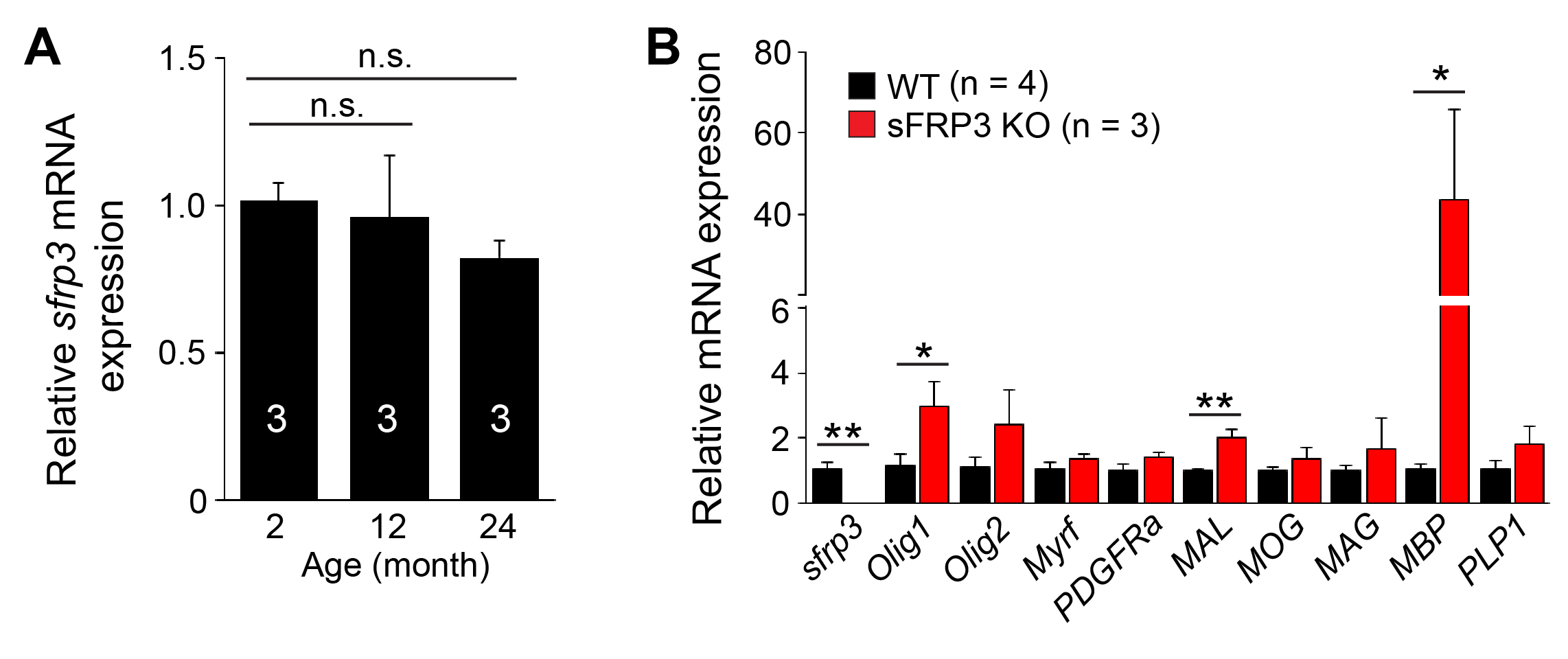


**SFig. 3. Genetic deletion of sFRP3 promotes myelin-related genes.**

(**A**) Quantification of *sfrp3* mRNA levels by quantitative real-time PCR (qRT-PCR). RNA was isolated from the hippocampus of 2, 12 and 24 months aged C57BL/6 WT female mice. Values were normalized to 2-month-old mice. The number associated with each bar graph indicates the number of animals examined. (**B**) Quantification of hippocampal mRNA expression of selected genes important for myelination in 2-month-old male sFRP3 KO and their WT littermates. Myelin basic protein (MBP), Olig1, and myelin and lymphocyte (MAL) were significantly increased in sFRP3 KO mice relative to their WT littermates. All values represent mean ± SEM (*: *P* < 0.05, **: *P* < 0.01, n.s. not significant; One-way ANOVA for **A**, and student’s *t*-test for **B**).

**Supplementary Table 1. Summary of qRT-PCR primers used in this study**

| **Target** | **Sequence** | **Product (bp)** |
| --- | --- | --- |
| mSFRP3-F | CAAGGGACACCGTCAATCTT | 182 |
| mSFRP3-R | CATATCCCAGCGCTTGACTT |  |
| mOilg1-F | CCACCACAACTCACCCACTG | 65 |
| mOilg1-R | ACGGATACGAGAATAGCCCG |  |
| mOlig2-F | CGCAGCGAGCACCTCAAATCTAA | 81 |
| mOlig2-R | CCCAGGGATGATCTAAGCTCTCGAA |  |
| mMyrf-F | TGGCAACTTCACCTACCACA | 160 |
| mMyrf-R | GTGGAACCTCTGCAAAAAGC |  |
| mPDGFRα-F | GGAAGGACTGGAAGCTTGGGGC | 154 |
| mPDGFRα-R | GAGATGAGGCCCGGCCCTGTGA |  |
| mMal-F | TCACACTGGATGCAGCCTACC | 71 |
| mMal-R | CAGGGCTTCCAGAACTGAGG |  |
| mMog-F | ATGAAGGAGGCTACACCTGC | 123 |
| mMog-R | CAAGTGCGATGAGAGTCAGC |  |
| mMag-F | AACCAGTATGGCCAGAGAGC | 133 |
| mMag-R | GTTCCGGGTTGGATTTTACC |  |
| mMbp-F | CCCGTGGAGCCGTGATC | 81 |
| mMbp-R | TCTTCAAACGAAAAGGGA |  |
| mPlp-F | GTATAGGCAGTCTCTGCGCTGAT | 201 |
| mPlp-R | AAGTGGCAGCAATCATGAAGG |  |
| mGapdh-F | ACCCAGAAGACTGTGGATGG | 171 |
| mGapdh-R | CACATTGGGGGTAGGAACAC |  |

**References**

Chehrehasa F, Meedeniya AC, Dwyer P, Abrahamsen G, Mackay-Sim A (2009). EdU, a new thymidine analogue for labelling proliferating cells in the nervous system. *J Neurosci Methods*. **177**, 122-130.

Choi CI, Yoo KH, Hussaini SM, Jeon BT, Welby J, Gan H, Scarisbrick IA, Zhang Z, Baker DJ, van Deursen JM, Rodriguez M, Jang MH (2016). The progeroid gene BubR1 regulates axon myelination and motor function. *Aging (Albany NY)*. **8**, 2667-2688.

Jang MH, Bonaguidi MA, Kitabatake Y, Sun J, Song J, Kang E, Jun H, Zhong C, Su Y, Guo JU, Wang MX, Sailor KA, Kim JY, Gao Y, Christian KM, Ming GL, Song H (2013). Secreted frizzled-related protein 3 regulates activity-dependent adult hippocampal neurogenesis. *Cell Stem Cell*. **12**, 215-223.

Yang Z, Jun H, Choi CI, Yoo KH, Cho CH, Hussaini SMQ, Simmons AJ, Kim S, van Deursen JM, Baker DJ, Jang MH (2017). Age-related decline in BubR1 impairs adult hippocampal neurogenesis. *Aging Cell*. **16**, 598-601.
